# Supplementary material for: DiagnoDating: diagnostics for dated phylogenies in microbial population genetics
Source: Mol Biol Evol. 2026 Apr 13;43(4):msag093. doi: 10.1093/molbev/msag093 (PMC13103887; doi:10.1093/molbev/msag093)
Supplement: msag093_Supplementary_Data [file msag093_supplementary_data.pdf]

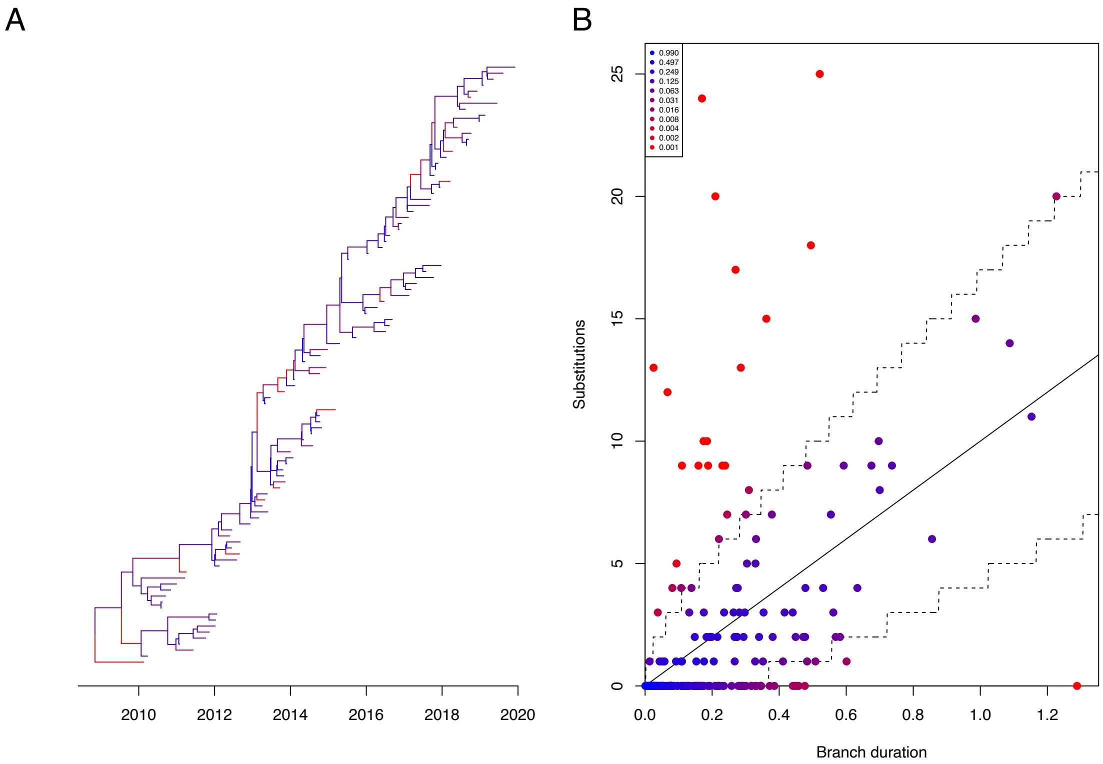

Figure S1: (A) Dated tree used as motivating example. (B) Likelihood of branches under a strict clock model with rate 10.

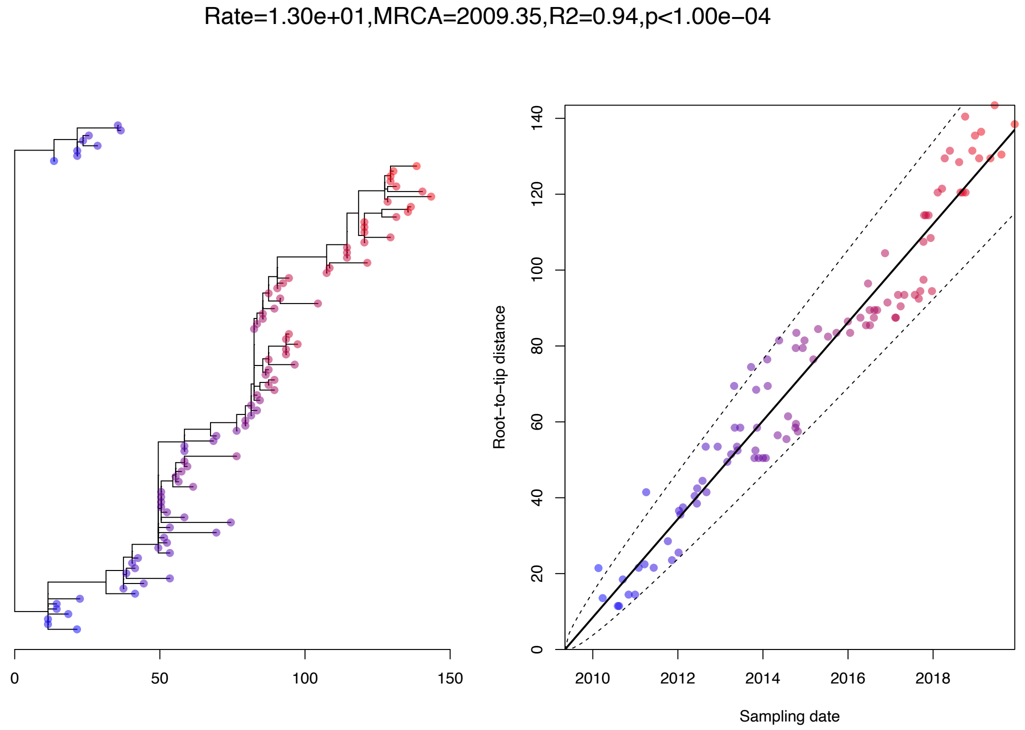

Figure S2: Root-to-tip regression analysis for the motivating example.

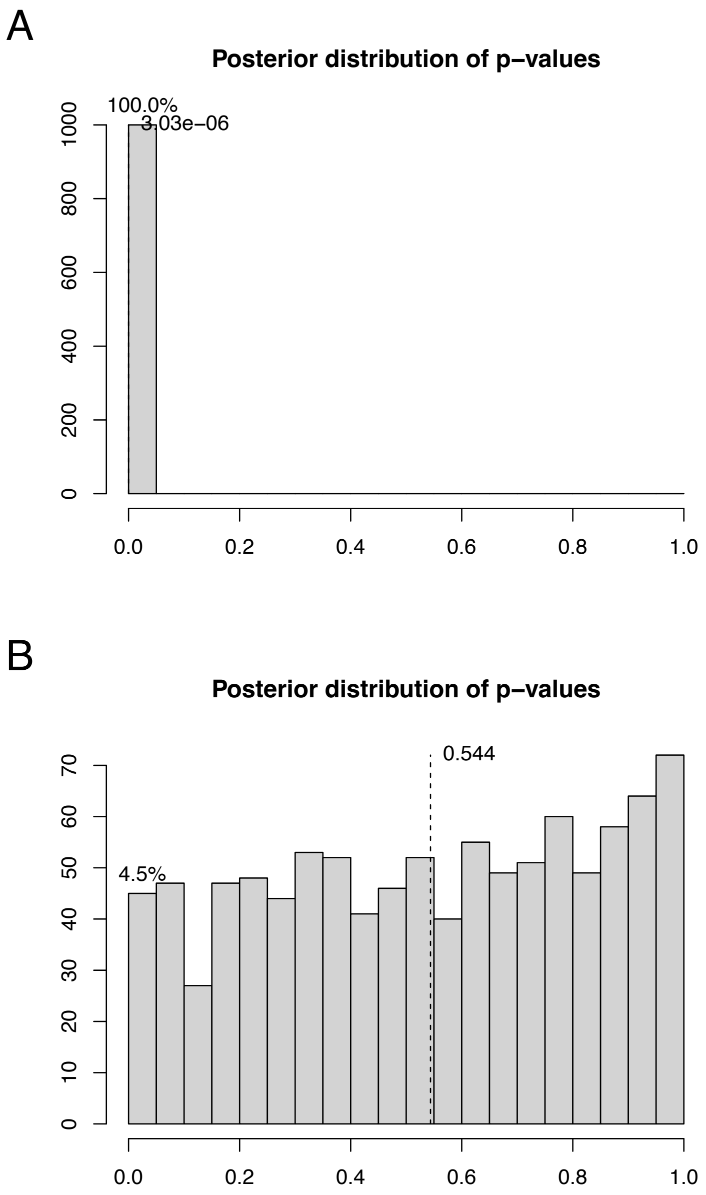

Figure S3: Posterior distribution of p-values for inference under the strict clock model (A) and ARC model (B).

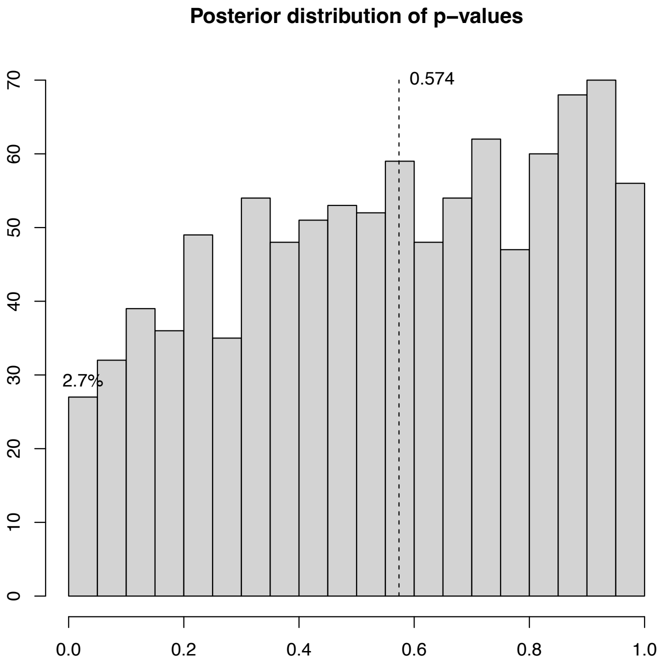

Figure S4: Posterior distribution of p-values for the pseudo-posterior based on ML inference.

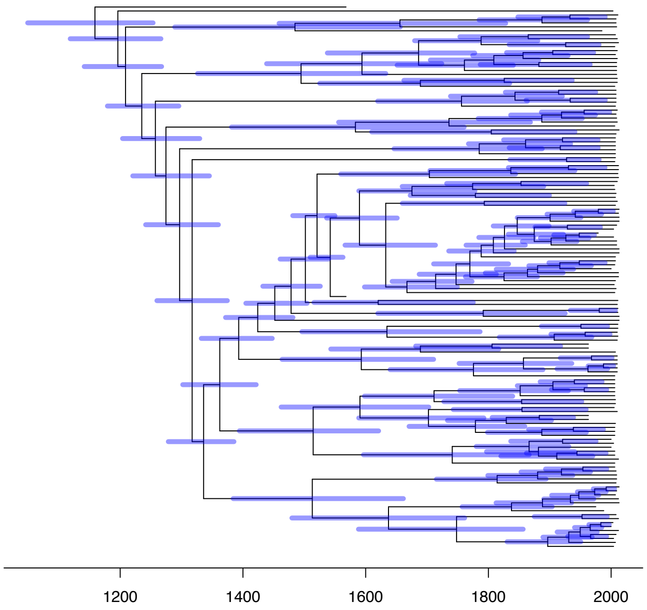

Figure S5: Dated phylogeny estimated for the HBV dataset.

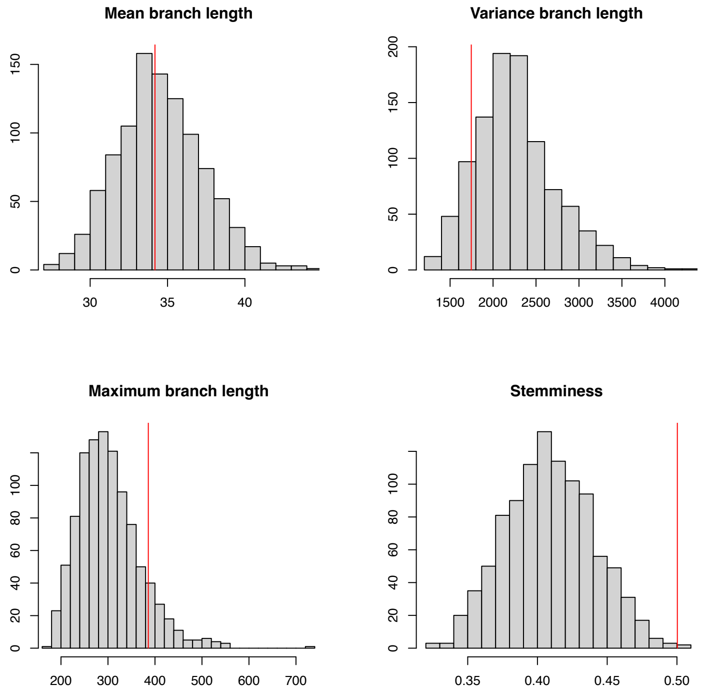

Figure S6: Posterior predictive analysis for the HBV application.

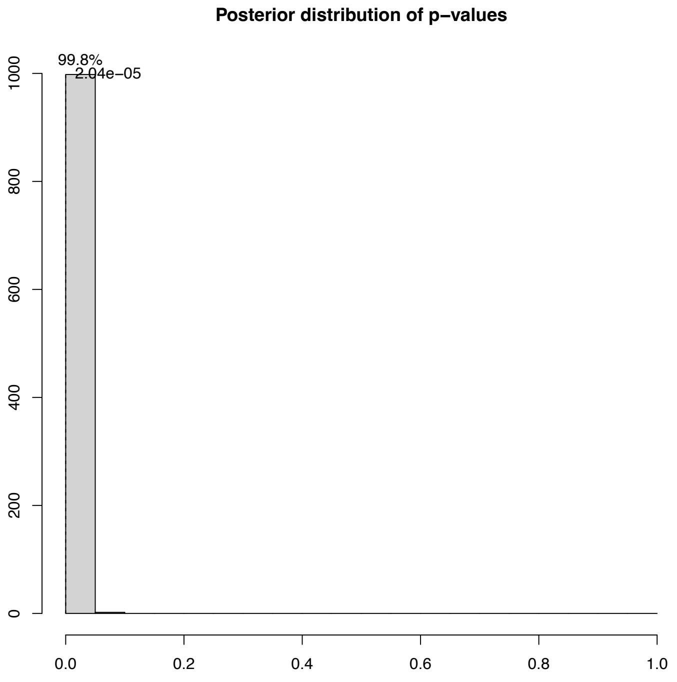

Figure S7: Posterior distribution of residual p-values for the HBV application.

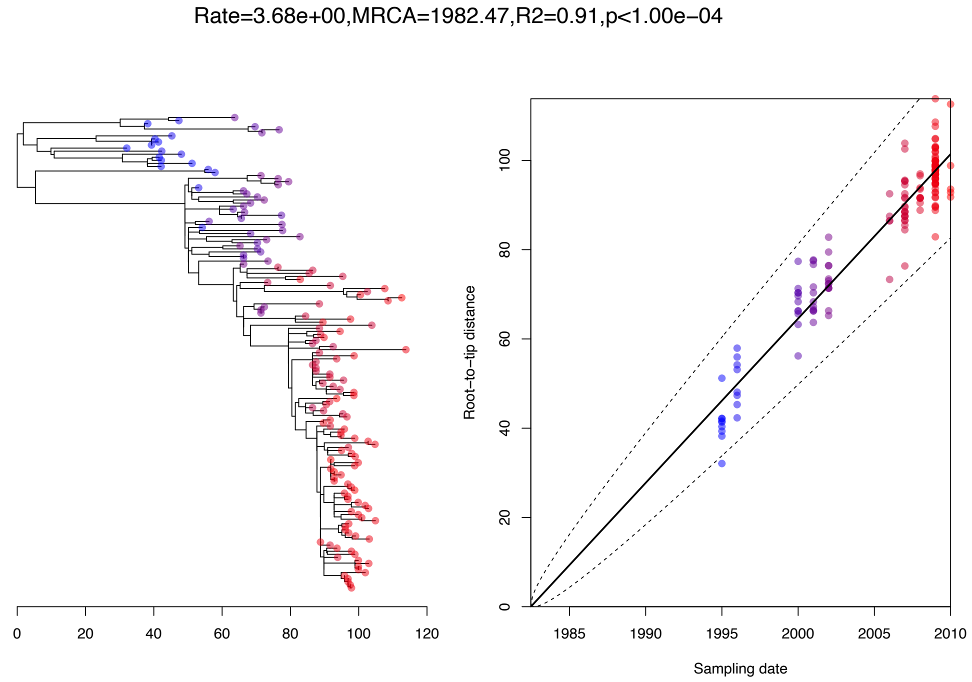

Figure S8: Root-to-tip analysis for the Shigella dataset.

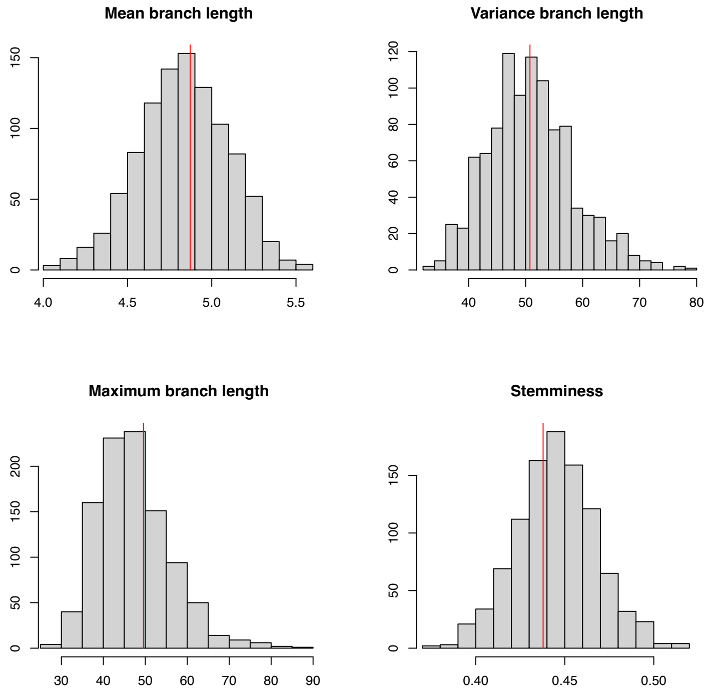

Figure S9: Posterior predictive analysis for the *Shigella sonnei* application.

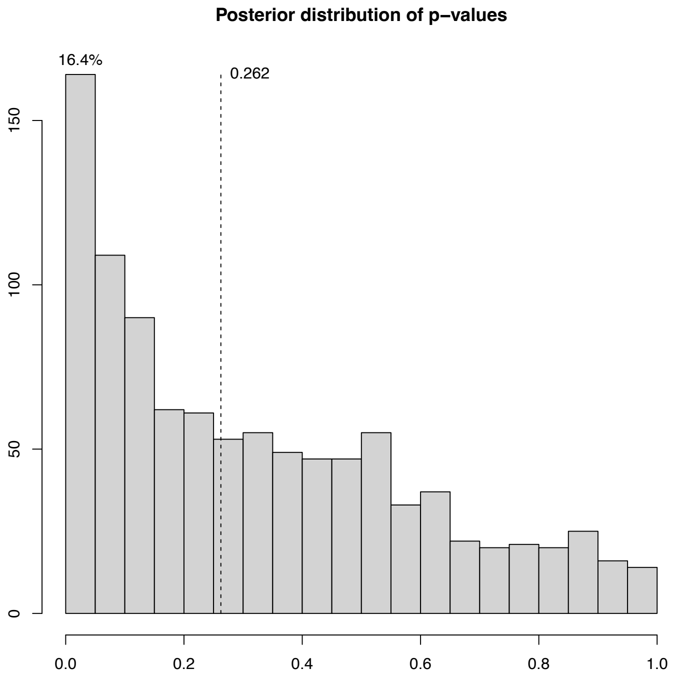

Figure S10: Posterior distribution of residual p-values for the *Shigella sonnei* application.

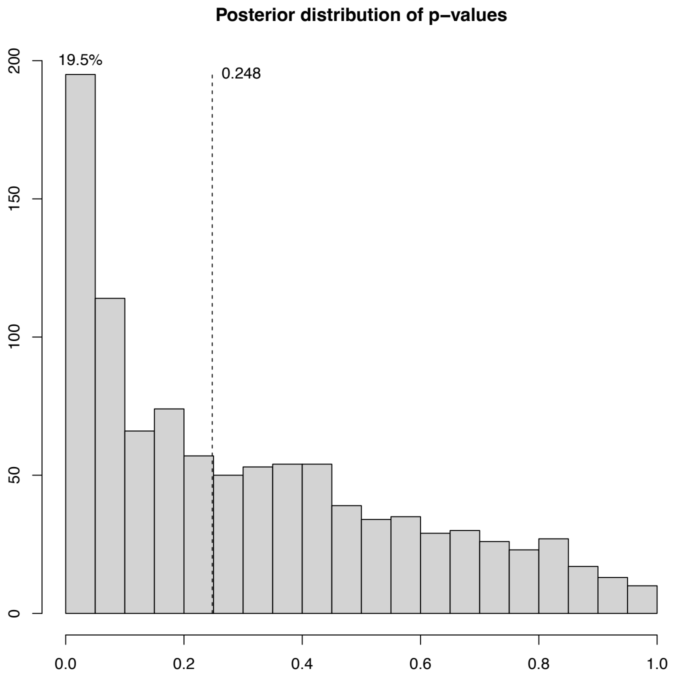

Figure S11: Posterior distribution of residual p-values for the PMEN1 application.

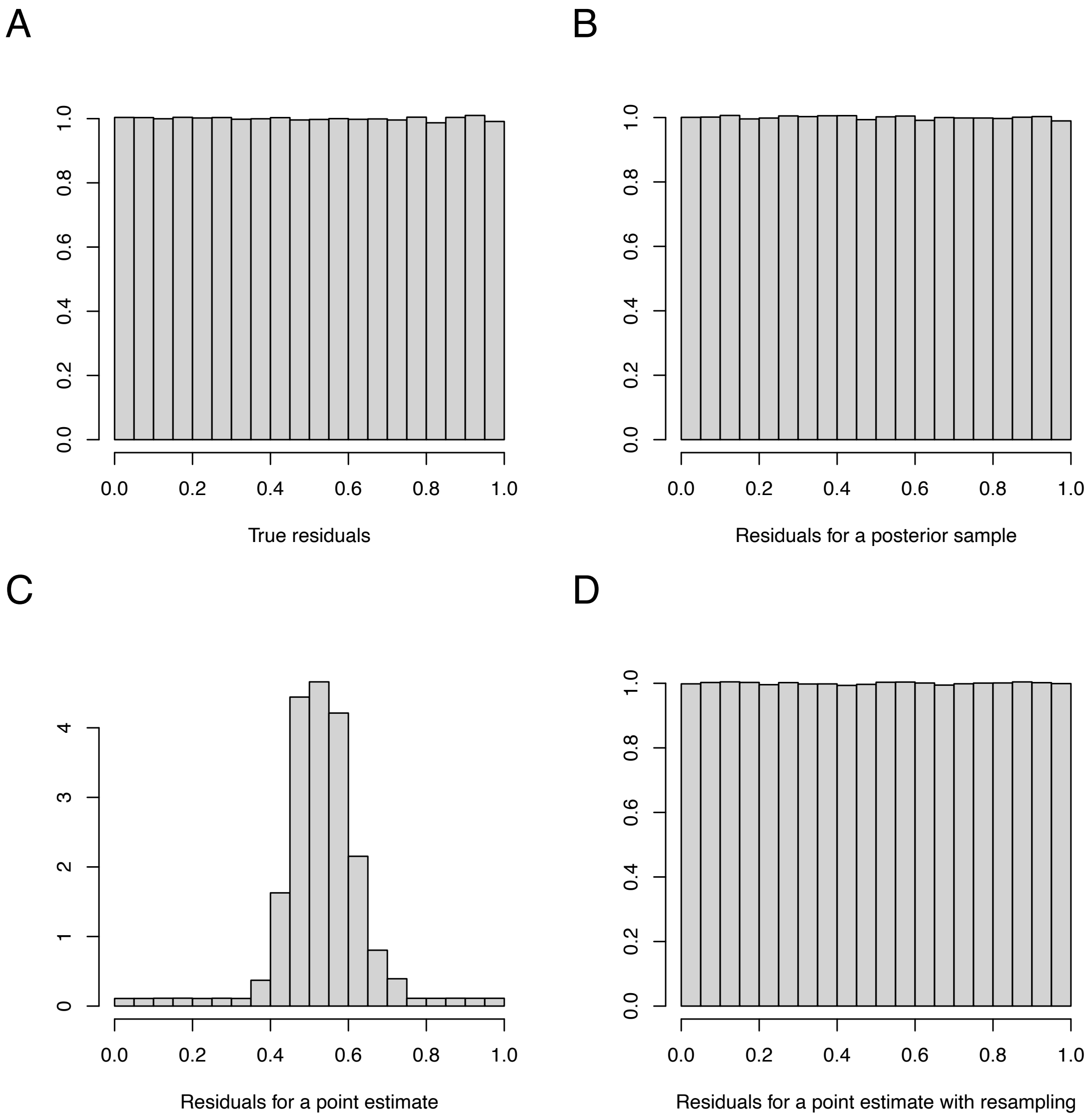

Figure S12: Uniform residuals in the independent and identically distributed case. (A) True residuals. (B) Residuals based on a posterior sample. (C) Residuals based on a point estimate. (D) Residuals based on a point estimate with resampling.
